# Supplementary figures and images for: Bacteria producing the bioplastic polyhydroxybutyrate kill the nematode Caenorhabditis elegans
Source: PLoS Biol. 2026 Apr 10;24(4):e3003748. doi: 10.1371/journal.pbio.3003748 (PMC13108865; doi:10.1371/journal.pbio.3003748)

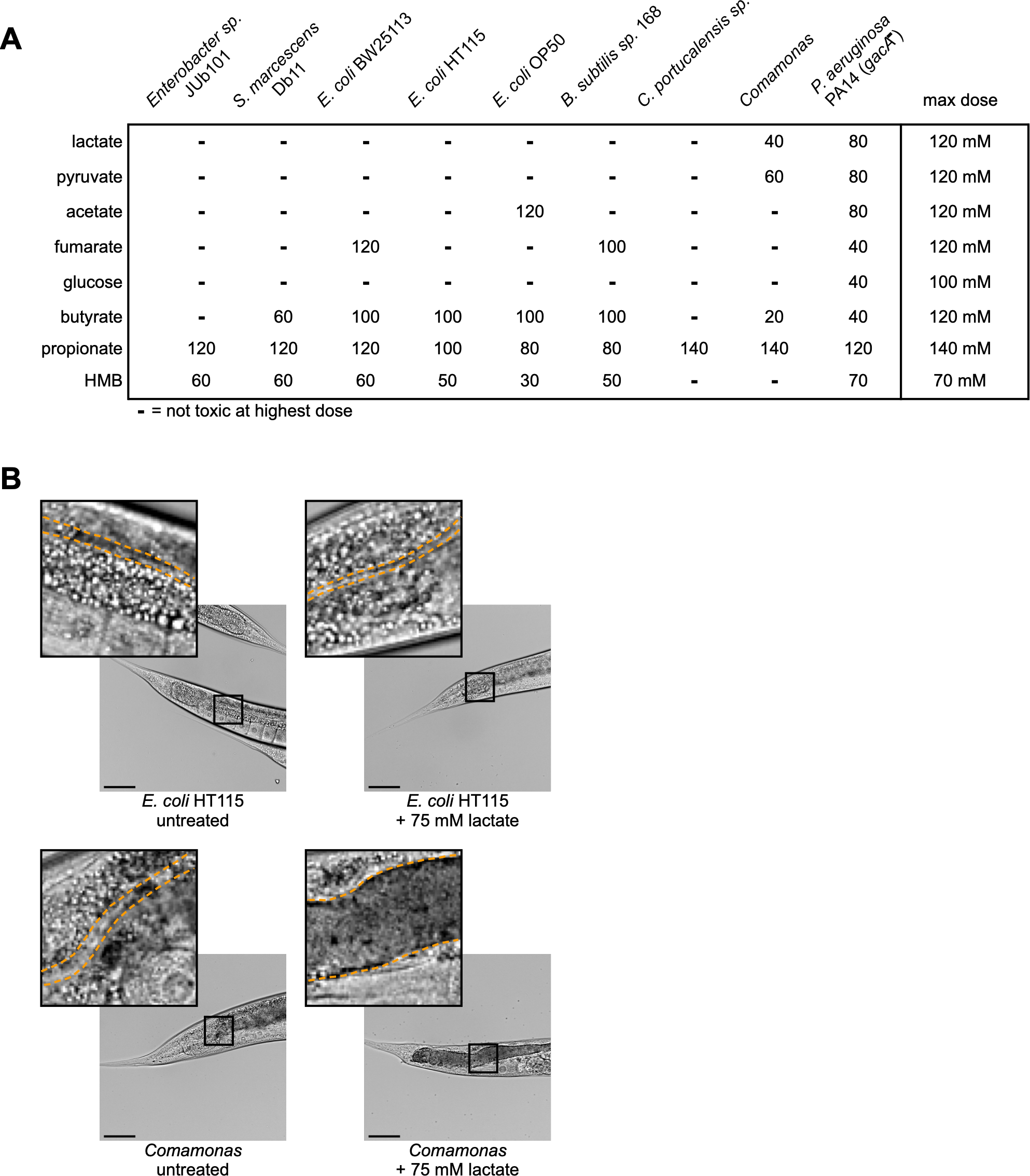

Supplement: S1 Fig — (A) MIC was determined qualitatively upon first observance of small or slow developing animals in the population. The data for this table can be found in S1 Data. (B) Brightfield images of adult animals. Orange dashed lines indicate intestinal lumen. Scale bar, 50 µm. (TIFF) [file pbio.3003748.s011.tiff]

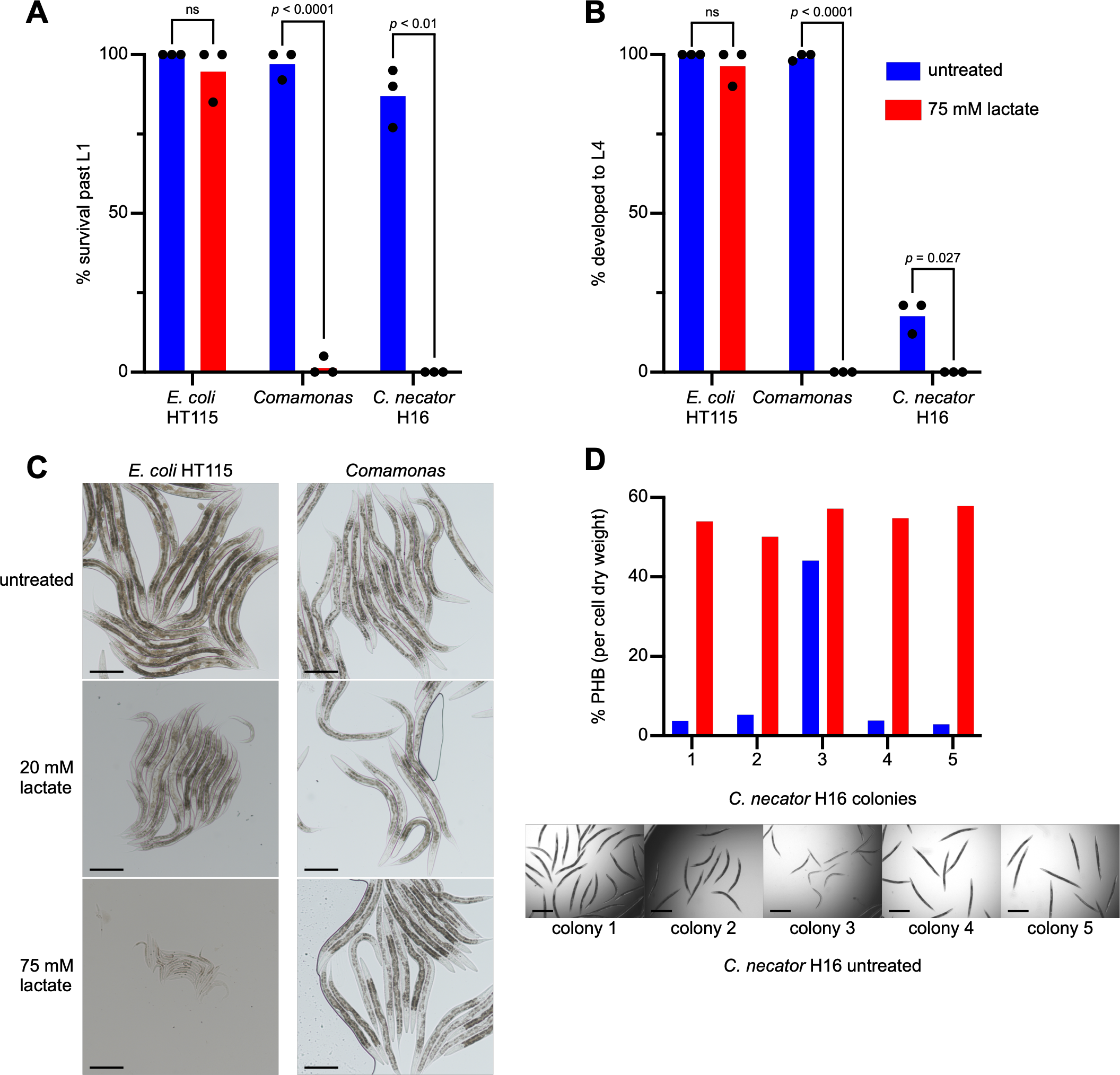

Supplement: S2 Fig — (A) Quantification of animal survival past the L1 stage. p values determined by unpaired Welch’s t test. (B) Quantification of animal development to the L4 stage. p values determined by unpaired Welch’s t test. (C) Brightfield images of adult animals grown on Comamonas plus/minus lactate. Scale bar, 100 µm. (D) GC-MS measurements of PHB from five different C. necator H16 colonies grown with or without 75 mM lactate. Brightfield images of animals grown on bacteria from the same C. necator colonies without lactate are shown below the bar graph. Scale bar, 500 µm. The data underlying this figure can be found in S1 Data. (TIFF) [file pbio.3003748.s012.tiff]

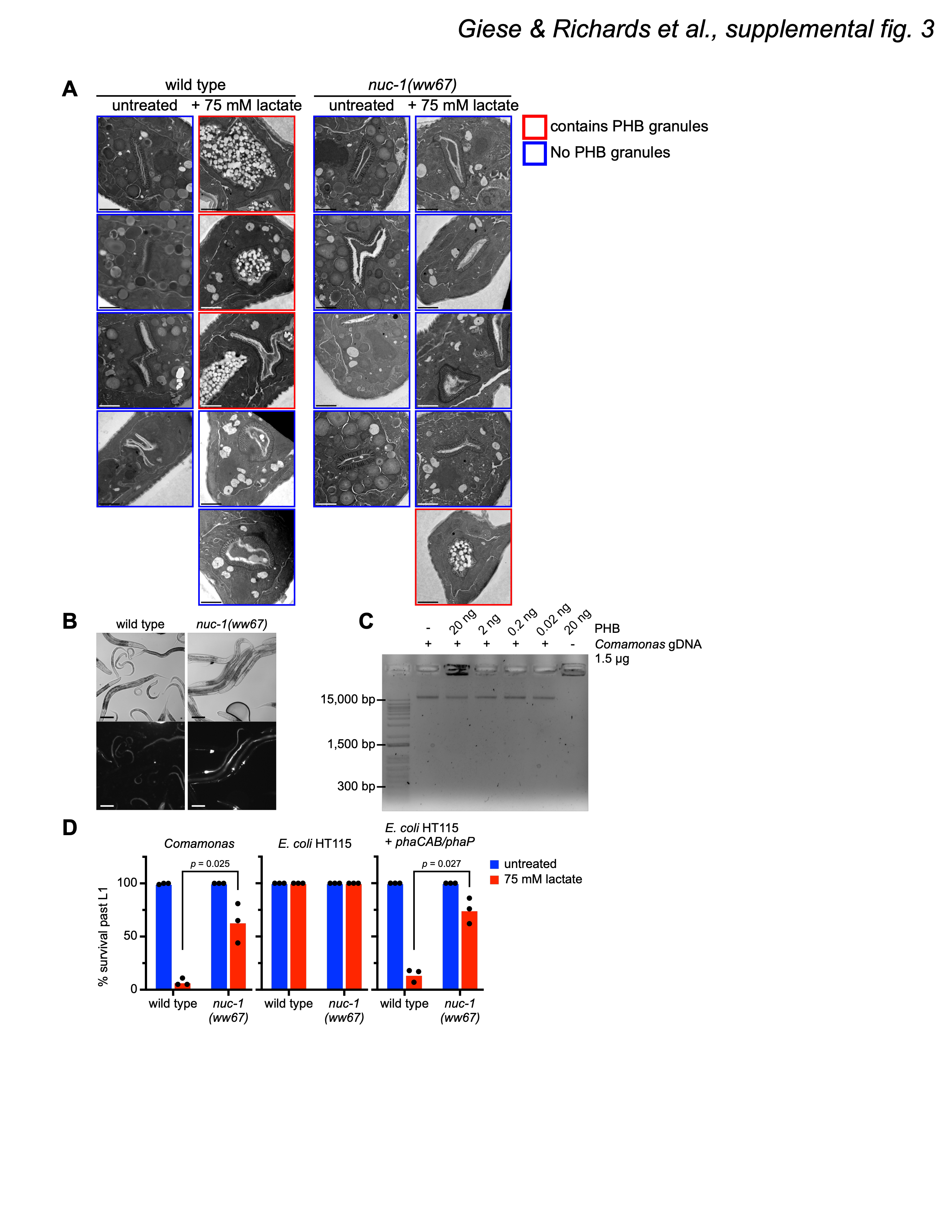

Supplement: S3 Fig — (A) Wild-type and nuc-1 mutant animals grown on Comamonas supplemented with lactate for 12 hours and imaged as cross-sections by TEM. Scale bar, 2 µm. (B) Phase and fluorescent images of mixed stage animals grown on Comamonas and stained with EtBr. Intensity of fluorescence seen along intestinal lumen indicated undigested gDNA. Scale bar, 100 µm. (C) One percent agarose gel with EtBr loaded with 50 µL PHB-gDNA mixture per well, where indicated. (D) Survival rate of wild-type and nuc-1 mutants grown on Comamonas, E. coli HT115, or E. coli HT115 + phaCAB/phaP with or without lactate. P values determined by two-tailed Welch’s t test. The underlying data for this graph can be found in S1 Data. (TIFF) [file pbio.3003748.s013.tiff]

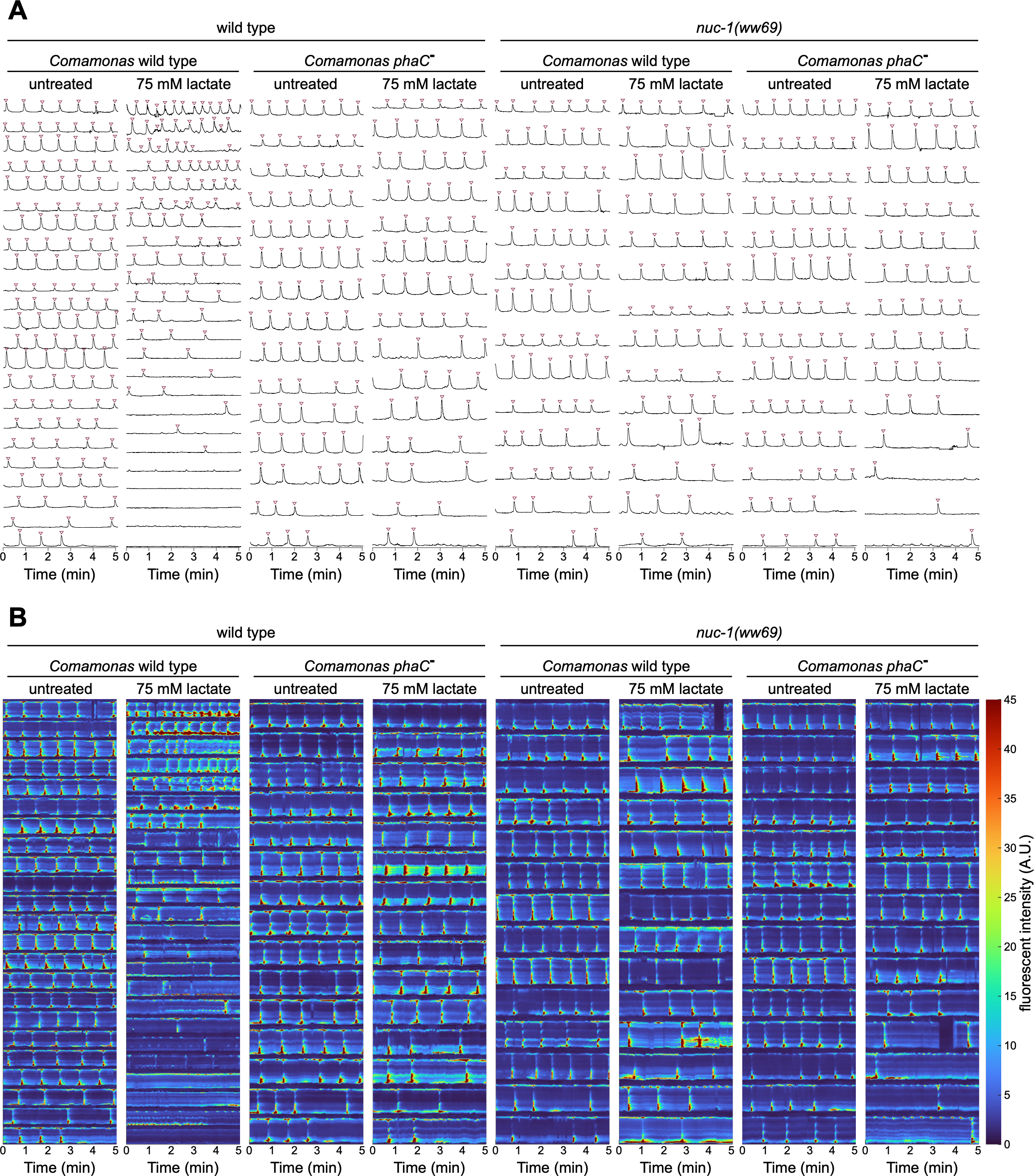

Supplement: S4 Fig — (A) Traces measuring mean intestinal GCaMP fluorescence in wild-type and nuc-1 mutants grown for 24-hours on Comamonas and the phaC mutant with and without 75 mM lactate. (B) Kymographs of intestinal GCaMP fluorescence from all animals measured of wild-type and nuc-1 mutants grown on Comamonas and the phaC mutant with and without 75 mM lactate for 24-hours. A.U., arbitrary units. The data underlying this figure can be found in S1 Data. (TIFF) [file pbio.3003748.s014.tiff]

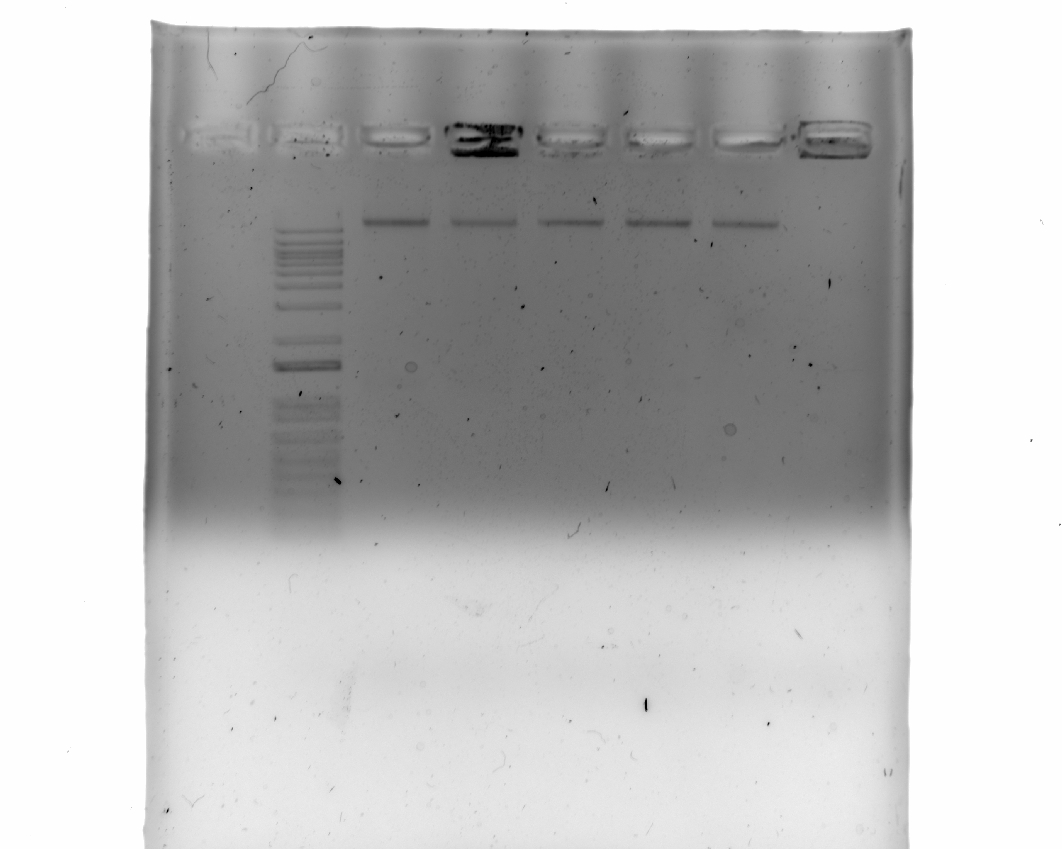

Supplement: S1 Raw Image — (JPG) [file pbio.3003748.s015.jpg]
