# Supplementary material for: Bacteria producing the bioplastic polyhydroxybutyrate kill the nematode Caenorhabditis elegans
Source: PLoS Biol. 2026 Apr 10;24(4):e3003748. doi: 10.1371/journal.pbio.3003748 (PMC13108865; doi:10.1371/journal.pbio.3003748)
Supplement: Attachment — Submitted filename: GIESE&RICHARDS_RESPONSE SUBMITTED.docx [file pbio.3003748.s018.docx]

We greatly appreciate the editors’ comments on our manuscript. We address them below (in blue) and hope that with the revisions we made, the manuscript is now acceptable for publication in PLoS Biology.

**Editorial Manager’s comments**

As I mentioned before, your manuscript has now been evaluated as Portable Peer Review from Cell by the editorial team in consultation with the Academic Editor. We find the experimental work strong and the evidence convincing that bacterial production of poly-β-hydroxybutyrate (PHB) is necessary and sufficient to induce lethality and physiological disruption in C. elegans.

However, as I mentioned in the invitation letter, we have concerns about how the findings are framed throughout the manuscript. As written, the title, abstract, Results headings, and parts of the Discussion give the impression that the study demonstrates toxicity of “bioplastics” or PHB itself. In contrast, the data support a more specific conclusion: that C. elegans dies when fed bacteria that produce PHB under the experimental conditions tested.

While no additional experiments are required; we do require substantial textual revisions to align the claims with the data.

In particular, we request:

- revision of the title to focus on PHB-producing bacteria rather than “bioplastics” broadly.

We would prefer to keep the word “*bioplastic*” in the title for a couple of reasons. First, PHB is itself a bioplastic and second, we think that this will draw the readers in more than PHB which most people may not be familiar with. We have, however, revised the title to: “*Bacteria producing the bioplastic polyhydroxybutyrate kill the nematode C. elegans”.* We hope this change will be satisfactory.

- removal or revision of the Abstract statement (lines 23–24) referring to “non-toxic” biodegradable bioplastics.

We have revised this statement in the abstract to better define PHB as a bioplastic and have removed the phrase “non-toxic”.

*“One such polymer, poly-β-hydroxybutyrate (PHB) is a biodegradable bioplastic.”*

- systematic adjustment of language throughout the Results and Discussion (e.g., line 170; lines 510–512) to avoid implying direct toxicity of purified PHB or commercial bioplastics, unless it is experimentally proven

We have changed the language throughout the manuscript to reflect the distinction between purified PHB and bacteria producing PHB.

- clear framing of broader environmental or biomedical implications as speculative rather than demonstrated

We have deemphasized speculation on the biomedical implications in the Discussion and focused more on the broad environmental implications. Lines 537-547 in the Discussion.

More specific *examples* and comments from the Academic Editor can be found at the end of this e-mail.

**Academic Editor’s comments**

Title: To me, the most important thing to change would be the title. I'd be OK with "bacterial production of poly-β-hydroxybutyrate kills the nematode C. elegans" or "poly-β-hydroxybutyrate-producing bacteria kill the nematode C. elegans" or along the lines what you had suggested as well.

Please see above.

Abstract: In the abstract, ideally, the sentence on bioplastics would be removed ("One such polymer, poly-β-hydroxybutyrate (PHB), is used commercially to produce ‘non-toxic’, biodegradable bioplastics."). At bare a minimum, to avoid the appearance that this study is about bioplastic toxicity, the word "non-toxic" should be removed.

As mentioned above we have removed the word “non-toxic” and adjusted the statement in the abstract.

Throughout the results: Replace "bioplastics", "bioplastic-producing bacteria", etc. with the specific compound name, i.e. "PHB" and "PHB-producing bacteria", etc.

We have made the adjustments throughout the text to clarify that the toxicity, as shown in the manuscript, arises from “PHB-producing bacteria” as opposed to “PHB” itself.

→ Other examples that can be used to consider the modifications:

Chaning names of subsections in the Results and Figures similar to "PHB kills C-elegans" for something similar to "C. elegans dies when bacteria it produces PHB"
